# Quantitative Investigation of the Rate of Intersystem Crossing in the Strong Exciton–Photon Coupling Regime

**DOI:** 10.1021/jacs.2c11531

**Published:** 2023-02-22

**Authors:** Arpita Mukherjee, Johannes Feist, Karl Börjesson

**Affiliations:** † Department of Chemistry and Molecular Biology, University of Gothenburg, Kemivägen 10, 412 96 Gothenburg, Sweden; ‡ Departamento de Física Teórica de la Materia Condensada and Condensed Matter Physics Center (IFIMAC), Universidad Autónoma de Madrid, Madrid E-28049, Spain

## Abstract

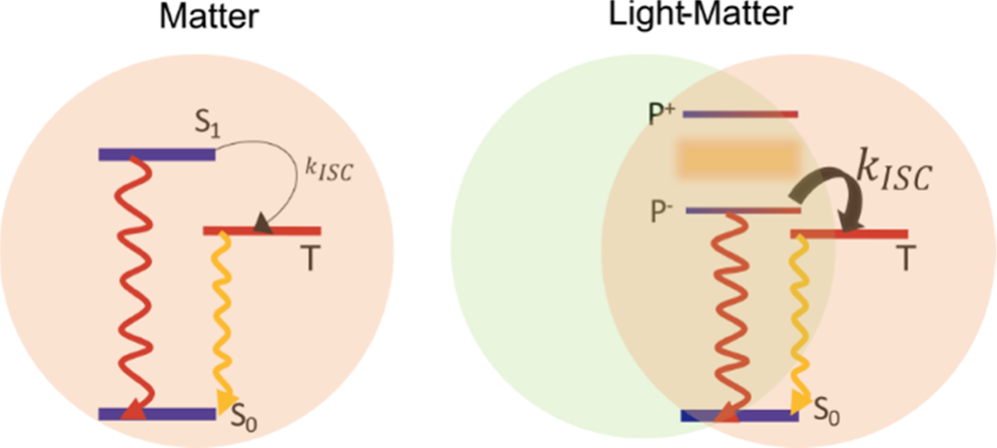

Strong interactions between excitons and photons lead to the formation of exciton-polaritons, which possess completely different properties compared to their constituents. The polaritons are created by incorporating a material in an optical cavity where the electromagnetic field is tightly confined. Over the last few years, the relaxation of polaritonic states has been shown to enable a new kind of energy transfer event, which is efficient at length scales substantially larger than the typical Förster radius. However, the importance of such energy transfer depends on the ability of the short-lived polaritonic states to efficiently decay to molecular localized states that can perform a photochemical process, such as charge transfer or triplet states. Here, we investigate quantitatively the interaction between polaritons and triplet states of erythrosine B in the strong coupling regime. We analyze the experimental data, collected mainly employing angle-resolved reflectivity and excitation measurements, using a rate equation model. We show that the rate of intersystem crossing from the polariton to the triplet states depends on the energy alignment of the excited polaritonic states. Furthermore, it is demonstrated that the rate of intersystem crossing can be substantially enhanced in the strong coupling regime to the point where it approaches the rate of the radiative decay of the polariton. In light of the opportunities that transitions from polaritonic to molecular localized states offer within molecular photophysics/chemistry and organic electronics, we hope that the quantitative understanding of such interactions gained from this study will aid in the development of polariton-empowered devices.

## Introduction

Exciton-polaritons are quasiparticles resulting from the strong interaction between excitons and an electromagnetic field.^1,2^ When an exciton couples to a resonant optical mode inside a cavity,^3,4^ a continuous reversible exchange of energy occurs, causing light–matter entanglement, and as a result, new hybrid light–matter states arise.^5,6^ Although these polaritonic states have contributions both from the exciton and the photon, the properties of the polaritons cannot be determined from a linear combination of the properties of its constituents.^7,8^ In addition, they show a dispersive behavior^9^ along with a delocalized nature^10−13^ due to their photonic character. Polariton formation has been demonstrated in various materials, e.g., inorganic semiconductors,^14−16^ and Rydberg atoms^17,18^ at low temperatures. Room-temperature polaritons have been observed experimentally in organic materials,^19^ followed by the emergence of unusual phenomena such as the formation of polaritonic Bose–Einstein condensates at room temperature,^20,21^ polariton lasing,^22−25^ and ultra-long-range energy transport.^26−31^ The reason behind the formation of stable room-temperature polaritons in organics is their low dielectric constants, which give rise to the formation of bound electron–hole pairs with a large binding energy, on the order of 0.5–1 eV. In addition, organic dyes also possess large transition dipole moments, which enhances the light–matter interaction.

**Figure 1:**
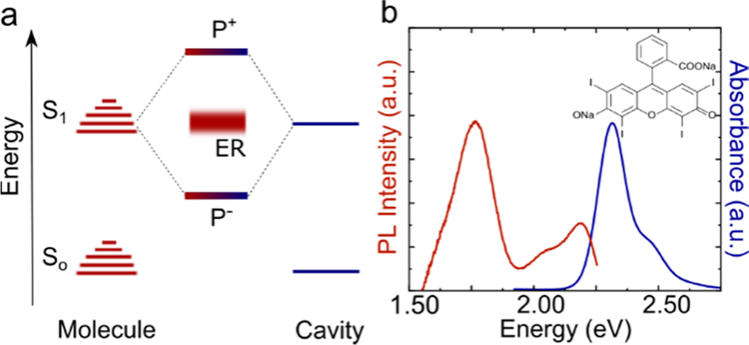
(a) Energy diagram showing how the molecular S_1_–S_0_ transition couples to an on-resonance cavity mode, forming two hybrid light–matter states (P^+^ and P^–^) and a set of optical dark states (ER). (b) Absorption (blue line) and emission (red line) spectra of a 15 wt % ErB film in a PVA polymer matrix (glass support/100 nm Ag/ErB-PVA film). The absorbance was calculated as *A* = log (1/*R*), assuming that the reflectivity from the 100 nm Ag mirror is 100%. The chemical structure of ErB is shown in the inset.

Although organic dyes are favorable candidates for strong exciton–photon coupling, they are not ideal two-level systems. Organic dyes can be involved in photochemical transformations^32−34^ and spin conversions^35^ to name a few excited-state processes. In an optical cavity containing a dye film, dyes are collectively coupled to the cavity mode. Ideally, if *N* molecules couple collectively to a single cavity mode, then two polaritonic states form, the upper (P^+^) and the lower (P^–^) polaritons (Figure 1a). These optically active hybrid states are delocalized over the entire cavity volume. The remaining *N* – 1 states form what is usually denoted as the exciton reservoir (ER). These molecular localized optically inactive states have an energy envelope resembling that of the bare molecular transition (S_1_). Although the relaxation dynamics in strongly coupled systems is heavily dominated by processes going through the exciton reservoir, it is not clear if it always governs excited-state relaxation efficiencies.^1,10,36−39^

Lately, the photochemistry of polaritonic states has been given large attention. For example, relaxation through the ladder of polaritonic states leads to a new kind of energy transfer process, which is efficient at length scales substantially larger than the typical Förster radius.^26−28,30,40−42^ Photochemical reactions, such as photoisomerization^13,43−45^ or photobleaching,^46,47^ in the strong coupling regime are mainly governed by the interaction between the delocalized polaritonic and the localized molecular centered states, and the yield of this processes varies with the extent of delocalization of the polaritonic state.^48,49^ Furthermore, transitions from molecular to polaritonic states have been studied by exploring reverse intersystem crossing (RISC)^50−52^ and triplet–triplet annihilation.^31,53,54^ However, the role of the exciton reservoir in several of these examples is under debate, specifically, if the photochemical processes occur directly from the polaritonic state or through the exciton reservoir. Increasing the understanding of the interaction between the polaritonic and molecular states is therefore of fundamental interest. This is because the scope of possibilities increases if the processes are predominantly going directly through the polaritonic state.

In this article, we report a systematic study of the rate of intersystem crossing (ISC) in the strong coupling regime. We used angle-resolved reflectivity and excitation spectroscopy to probe the relaxation pathways from the polaritonic to the triplet state of the molecule. A rate equation model was used to quantitatively analyze the experimental data under systematic experimental variations. The results indicate the presence of a direct transfer from the initially excited polaritonic state to the first excited triplet state and further how such a transfer varies with the energy alignment of the involved states. The study elucidates a quantitative understanding of the interactions between polaritonic and triplet states, which will ultimately aid in the development of polariton-empowered devices.

## Results and Discussion

### System under Study

To explore the effect of strong exciton–photon coupling on the rate of intersystem crossing (ISC), an organic molecule having a moderate ISC yield and the ability to enter the strong coupling regime is needed. Erythrosine B (ErB), the tetraiodized derivative of fluorescein, was chosen as the model system as it has already been used to study polariton–triplet state interactions in the strong coupling regime.^52^ The absorbance and emission spectra of an ErB film (15 wt % in PVA) are shown in Figure 1b. The film was made by spin-coating a water solution containing ErB and poly(vinyl alcohol) (PVA) on top of a 100 nm thick Ag mirror deposited on a glass substrate. Figure 1b shows that the absorbance is centered at 2.3 eV with a small shoulder around 2.5 eV. The fluorescence and phosphorescence are centered at 2.2 and 1.8 eV, respectively. The occurrence of both fluorescence and phosphorescence together with the sharp and strong absorption suggests that we can probe the ISC process of this system in the strong coupling regime at room temperature. As a note on the use of ErB, during this study, we found that the photophysics of ErB changes in an unpredictable manner with concentration. This molecule is therefore not suitable for concentration-dependent studies,^52^ and consequently, we consistently use the same concentration of ErB throughout this study.

### Entering the Strong Coupling Regime

**Figure 2:**
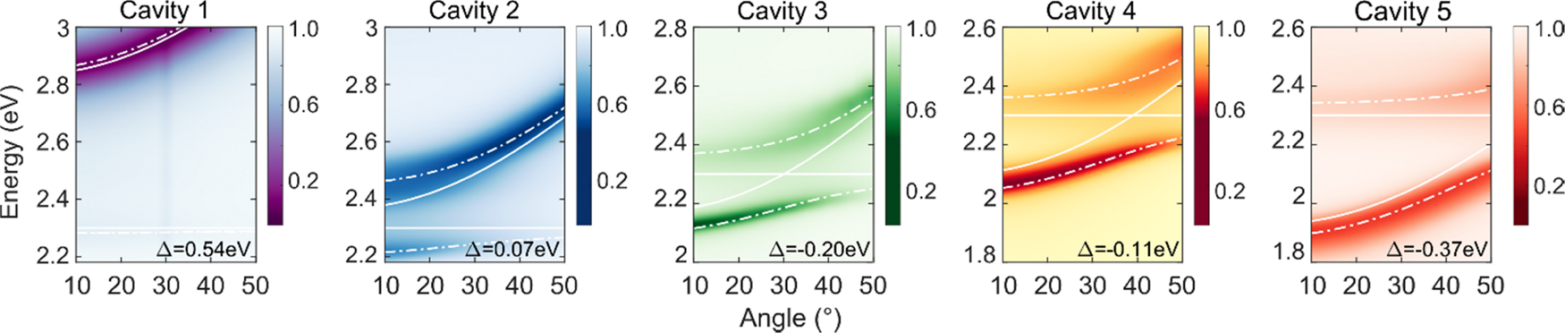
Normalized angle-dependent reflectivity spectra, collected in TE mode, of five different cavities. In all cases, the data are fitted with a coupled harmonic oscillator model (polariton dispersion is represented by the white dashed line, and the nondispersive ErB absorption (*E*_x_) and cavity dispersion (*E*_c_) are shown in white lines). The energy detuning of each cavity is shown as Δ.

To enter the strong exciton–photon coupling regime, it is important to have a discrete photonic mode that strongly couples to the molecular transition. The necessary field confinement was obtained using a Fabry–Pérot cavity, into which ErB was introduced. The cavities were formed by sandwiching a film containing ErB within a PVA polymer matrix in between two Ag mirrors, and the cavity resonance frequency was controlled through the film thickness. To facilitate probing of the system, one of the mirrors was made thick enough (100 nm) to reflect all light, while the other was semitransparent (30 nm). The absorbance of a reference cavity containing no dye molecules (glass support/100 nm Ag/PVA film/30 nm Ag) is given in Figure S1. Our study is concentrated on five different cavities, having different thicknesses and thus different coupling parameters. The details of the parameters related to the cavities are given in Table S1. To ensure that all of the cavities have entered the strong coupling regime, angle-resolved reflectivity spectra of the cavities were measured. These spectra (measured in TE mode) are shown in Figure 2. Inside the cavities, the molecular absorption splits into two polaritonic branches, P^+^ and P^–^. To obtain the coupling parameters of each cavity, the experimentally obtained polariton energies were fitted with a coupled harmonic oscillator model^6^
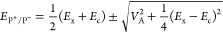
1where *E*_P^+^_ and *E*_P^–^_ are the energies of P^+^ and P^–^, respectively, *E*_x_ is the exciton energy, *E*_c_ is the angle-dependent cavity energy, and *V*_A_ is the light–matter coupling strength. The fitting parameters and the exciton–photon fractions of the P^–^ branch are given in Table S2 and Figure S2, respectively. The exciton–photon coupling strength of each cavity is larger than energy dissipation, suggesting that they are all within the strong coupling regime. Here, the five cavities represent a detuning series, from the cavity mode being at a considerably higher energy than the exciton energy (cavity 1, most blue detuned or even off-tuned) all the way to the cavity mode (at normal incidence) being at a considerably lower energy than the exciton (cavity 5, most red-detuned). Thus, with these cavities, we can examine if the intersystem crossing behavior changes with cavity–exciton energy detuning.

### Angle-Resolved Excitation

To understand the ISC process of ErB in the strong coupling regime, we study the excited-state relaxation pathways of the polaritons. By comparing the excitation and absorption spectra, the relaxation efficiency of higher excited states can be examined. We measured the excitation and absorption spectra as a function of incident angle. The emission was monitored about 20 nm toward longer wavelengths as compared to the P^–^ emission maximum and monitored at 20° in the orthogonal plane compared to the angle of the incident light to avoid specular reflection into the detector (Scheme S2). As we are interested in the ISC process, we measure the spectra both in the prompt (within a few ns after excitation) and delayed (after tens of μs after excitation) regimes. The prompt excitation mainly gives an overview of the relaxation from the polaritonic state just after excitation (Scheme S3). On the other hand, when the system is probed in the delayed regime, only long-lived states such as triplet states persist. As a consequence, the delayed excitation spectra predominantly probe the relaxation from the lower polariton branch and exciton reservoir to triplet states. Note that in this study, we are monitoring the emission from P^–^; the delayed excitation spectra therefore also involve either reversed intersystem crossing (RISC) or radiative pumping of P^–^ from the triplet states. However, as we probe at a constant emission angle, both of these processes are expected to be the same regardless of the excitation angle, and as a result, the delayed emission can be considered to be proportional to the triplet state concentration. Further, the excitation and emission angles relate to horizontal and vertical directions, respectively. Excitation and emission at 20° thus relate to different in-plane momenta (although with the same absolute value), and specular reflection was therefore avoided in the experiments. With the Ag mirror thicknesses used, we also observe phosphorescence, but the intensity is much lower as compared to P^–^ emission in the delayed regime (Figure S3). Monitoring P^–^ therefore gives experimental data with the largest signal-to-noise ratio.

**Figure 3:**
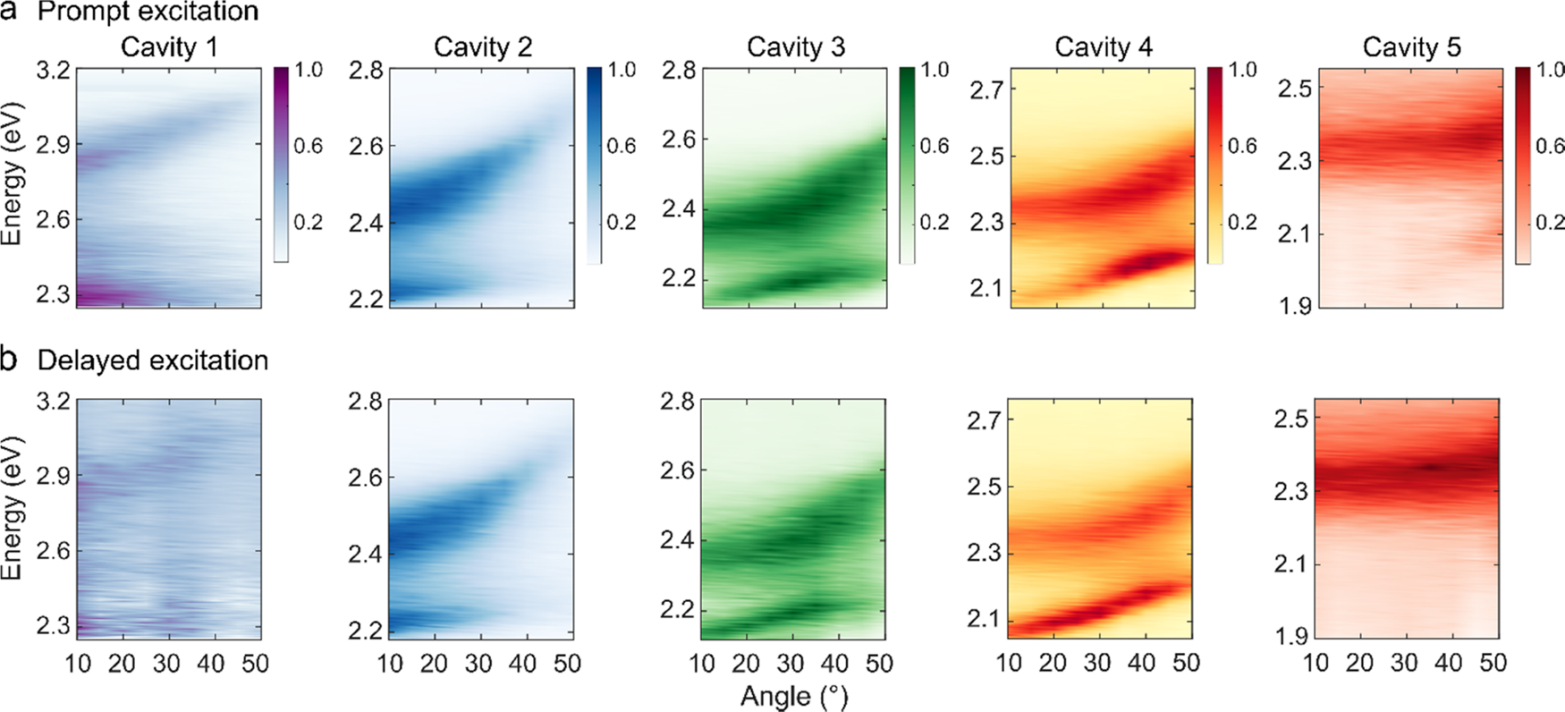
Excitation spectra as a function of excitation angle in the (a) prompt and (b) delayed regimes (collected in TE mode). In the measurements, the excitation and the emission paths were fiber-coupled to the rotating arm of a goniometer, and the excitation angle was changed in 5° intervals while maintaining the emission angle constant, at 20° in the orthogonal plane. The emission was collected at 2.194, 2.101, 2.066, 2.000, and 1.851 eV for cavities 1–5, respectively. The data are normalized.

The normalized angle-resolved prompt and delayed excitation spectra (collected in TE mode) for all of the cavities are shown in Figure 3a,b, respectively. In cavities 1 and 2, the intensity of the prompt and delayed excitation spectra varies in a similar fashion with the excitation angle in both the P^+^ and P^–^ branches (Figure 3). However, with increasing photonic contribution of P^–^, this is no longer true for the P^–^ branch (cavities 3, 4, and 5). Now, the intensity of the delayed and prompt emission varies differently with the excitation angle. This is most pronounced in cavity 4, where excitation of the P^–^ branch at low angles results in a relatively larger delay compared to prompt emission, thus indicating that a new relaxation pathway toward the triplet states is active in these cavities. To confirm that our experimental findings are not due to any intrinsic property of the molecule, the prompt and delayed excitation spectra of the bare film were measured (Figure S4). The data suggest that the experimental results obtained in Figure 3 are not due to an intrinsic property of the molecule; thus, the effect is generated due to polariton dynamics. In addition, as the emission is monitored at a constant angle while recording the excitation spectra in Figure 3, we also measured the angular resolved emission (Figure S5). The emission from all cavities changes in a similar way with angle, and as a result, we can treat all of the cavities in a similar manner.

### Relaxation Efficiency

**Figure 4:**
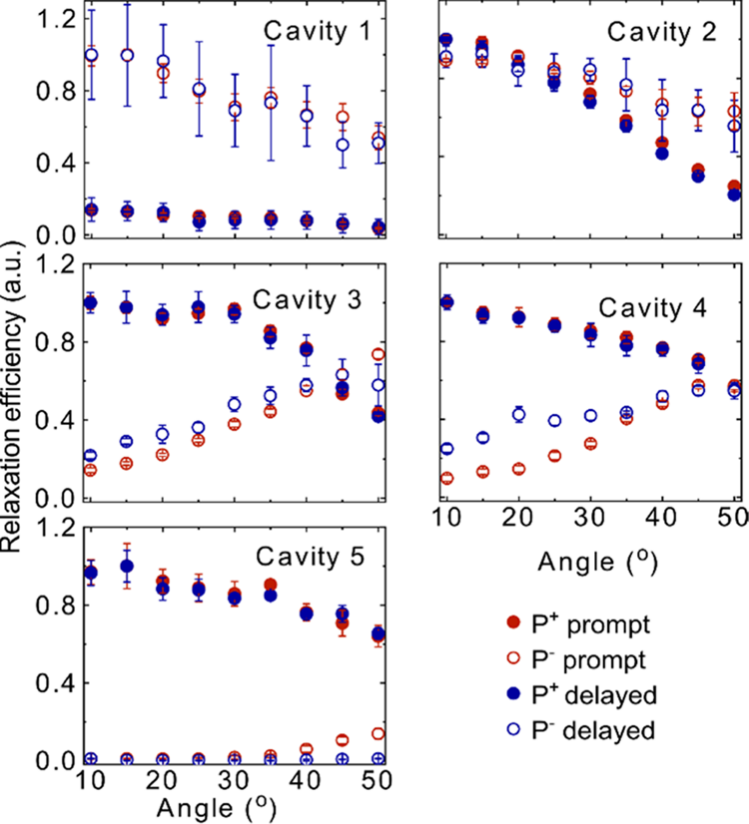
Relative relaxation efficiency of the upper and lower polariton branches of all of the cavities. The red closed and open circles represent the efficiency in the prompt regime and the blue closed and open circles represent the efficiency in the delayed regime for P^+^ and P^–^, respectively. The data are normalized at the maximum efficiency. The error bars represent the standard deviation of the experimental result from the mean value.

To understand the relaxation pathways in a more detailed manner, we next study the relative prompt and delayed relaxation efficiencies of each branch. For that, the excitation spectra are divided by the absorption at each angle. This type of analysis has previously been done to probe energy relaxation toward the lowest in-plane momentum states of the P^–^ branch in organic microcavities.^26,55,56^ The relative relaxation efficiencies are presented in Figure 4 for all of the cavities (see Figures S6 and S7 for the corresponding excitation intensity and absorption, respectively). In blue detuned cavities, the relaxation efficiency from P^+^ (red and blue dots) is lower compared to that of P^–^ (red and blue open circles). A gradual change in this ratio is seen as the cavity energy decreases through the series. As the relaxation efficiency from P^+^ is mainly guided by the relaxation from P^+^ to the exciton reservoir, the relaxation efficiency depends on the exciton fraction of P^+^ as well as the energy difference between the exciton reservoir and P^+^. In a blue detuned cavity, P^+^ is less excitonic and energetically further away from the exciton reservoir (Figure 2). Energy relaxation thus becomes less probable, which is experimentally seen as a relatively less-efficient relaxation pathway from P^+^. In addition, the normalized delayed and prompt relaxation efficiencies from the P^+^ branch are the same for all of the cavities. This behavior is quite intuitive as the P^+^ population relaxes through the exciton reservoir, where the in-plane momentum information is lost in both the prompt and delayed regimes. The reason for the loss of in-plane momentum is because the reservoir states do not have a well-defined momentum. Furthermore, at the dye loading concentrations used, intermolecular energy transfer is faster than the exciton reservoir lifetime. Thus, any anisotropic population distribution rapidly becomes randomized. On the contrary, in cavities having a large photonic fraction in P^–^ (cavities 3, 4, and 5), the relaxation efficiency from P^–^ does not show the same angular dependence of the relaxation efficiency in the prompt and delayed regimes. Here, different relaxation pathways within the prompt and delayed regimes must be involved. In addition, the delayed relaxation efficiency of P^–^ of cavity 5 is negligible. The reason is that, as cavity 5 is largely red-detuned, the P^–^ state possesses a mostly photonic character and therefore scatters most of the incident light, which can also be visible in the prompt relaxation efficiencies (red open circles in Figure 4, cavity 5). Thus, at lower angles, the prompt relaxation efficiency is negligible, but it becomes prominent at a higher excitation angle due to an enhancement of the excitonic character. It is important to mention that, during the calculation of the relaxation efficiency, the Ag mirror absorption was considered negligible. This is a valid approximation as the mirror absorption is very much smaller compared to the absorption of an ErB dye film (Figure S9).

### Rate Equation Model

**Figure 5:**
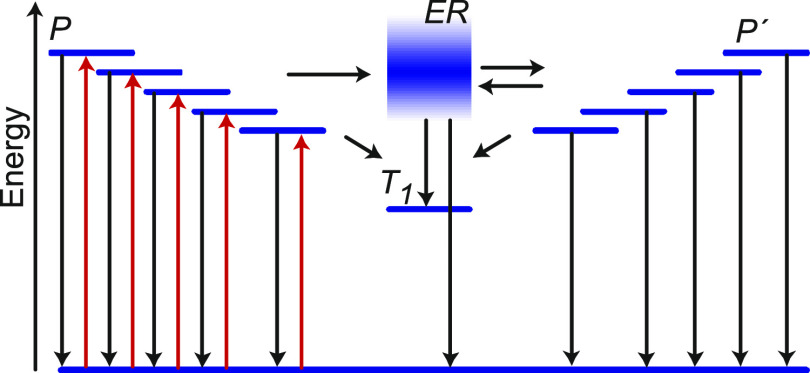
Schematic illustration showing the states (P, P′, ER, and T_1_) involved in modeling (blue) together with the processes occurring. The different excitation angles are displayed as excitations (red arrows) to different polaritonic states. Processes considered in the model are displayed as black arrows. Note that for a lack of space, only 5 out of the 9 polaritonic states are used in the model shown in the figure (corresponding to 10–50° at a 5° interval). Thus, for each excitation angle, one P state, one ER state, one T_1_ state, and nine P′ states are taken into account. In the simulations, all nine excitation angles were fitted globally.

We will now explore how the difference in the relaxation efficiency when exciting P^–^ between the prompt and delayed regime in red-detuned cavities can be quantitatively explained. We focus on cavity 4 as it shows the largest difference between the prompt and delayed emission when exciting the P^–^ branch at different angles, i.e., the open red and blue circles for cavity 4 in Figure 4 do not overlap. It further has a larger energy separation between ER and P^–^, giving quantum mechanically more defined states. A model based on rate equations was built, which describes the population and depopulation of P^–^ and the exciton reservoir after exciting P^–^. The involved energy states and transitions in this model are depicted in Figure 5. Here, the ground state and the exciton reservoir are denoted as S_0_ and ER, respectively, and the triplet state by T_1_, and a set of P^–^ states having in-plane momenta dictated by the used excitation angles (shown in 10° intervals) are also shown (denoted as P). The population from all of these P states transfer to the same exciton reservoir, where the in-plane momentum is randomized. The population can now transfer to the P^–^ state with an in-plane momentum corresponding to the emission angle (denoted as P′). Although we are only monitoring the P′ emission at 20°, our model considers the reversible transfer between the exciton reservoir and a larger selection of P′ states (with in-plane momenta corresponding to the 10–50° interval) to correctly capture all of the processes that involve the lower polariton branch. It should be noted here that the excitation and emission angles relate to horizontal and vertical directions, respectively. Excitation and emission at 20° thus relate to different in-plane momenta (although with the same absolute value), and specular reflection was therefore avoided in the experiments.

After photoexcitation of P (Figure 5), the polariton population will decay through emission of a photon with a preserved in-plane momentum with rate constant *k*_r_, transfer to the exciton reservoir with rate constant *k*_P^–^ → ER_, or transfer to T_1_ with a rate constant *k*_P^–^ → T1_. It should be noted that the emitted photons at this point will not be experimentally observed. This is because the emission is measured at an angle that is different from the angle of excitation. The excited-state population in the exciton reservoir will decay to the ground state with rate constant *k*_nr_, transfer to T_1_ with rate constant *k*_ER → T1_, or transfer back (*k*_ER → P^–^_) and forth (*k*_* *P^–^ → ER_) to P′. The P′ population can also decay radiatively with rate constant *k*_r_ or transfer to T_1_ with rate constant *k*_P^–^ → T1_. The mathematical formulations of all rate constants are described in the Supporting Information (Section 2.6).

The time-dependent populations of the initially excited polaritonic state, P(θ, *t*), the exciton reservoir, ER(θ, *t*), and the generated polaritonic states after relaxation from ER, P′(θ, *t*), as a function of the excitation angle (θ), can be expressed as
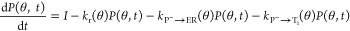
2
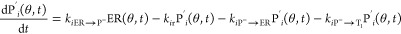
3
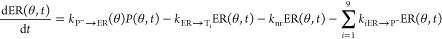
4where *I* is a constant excitation fluence. In eqs 3 and 4, *i* varies from 1 to 9, which signifies the angles 10–50° of the P′ states (the same interval as for the excitation angles). For each excitation angle, the model thus constitutes of one initially excited polariton state (P), the exciton reservoir, and a set of nine polariton states (P′).

The quantum yield of each decay route can now be described in the form of rates. At long time scales (steady-state conditions), the sum of all decay rates equals to the influx (*I*). The quantum yield of prompt emission (Φ_F_) at the monitored emission angle then can be expressed as
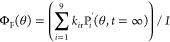
5

In the same manner, the quantum yield of the ISC process (Φ_ISC_) can be described as
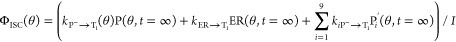
6

We employed this model to globally fit the experimental prompt and delayed relaxation efficiencies when exciting P between 10 and 50° in 5° intervals. However, first, the relative relaxation efficiencies shown in Figure 4 need to be renormalized to the absolute values. For that, the efficiencies were factorized with respect to the quantum yield of the prompt and delayed part of the emission. The details of this calculation and how the delayed quantum yield was related to the yield of ISC are given in the Supporting Information (Sections 2.8 and 2.9).

**Figure 6:**
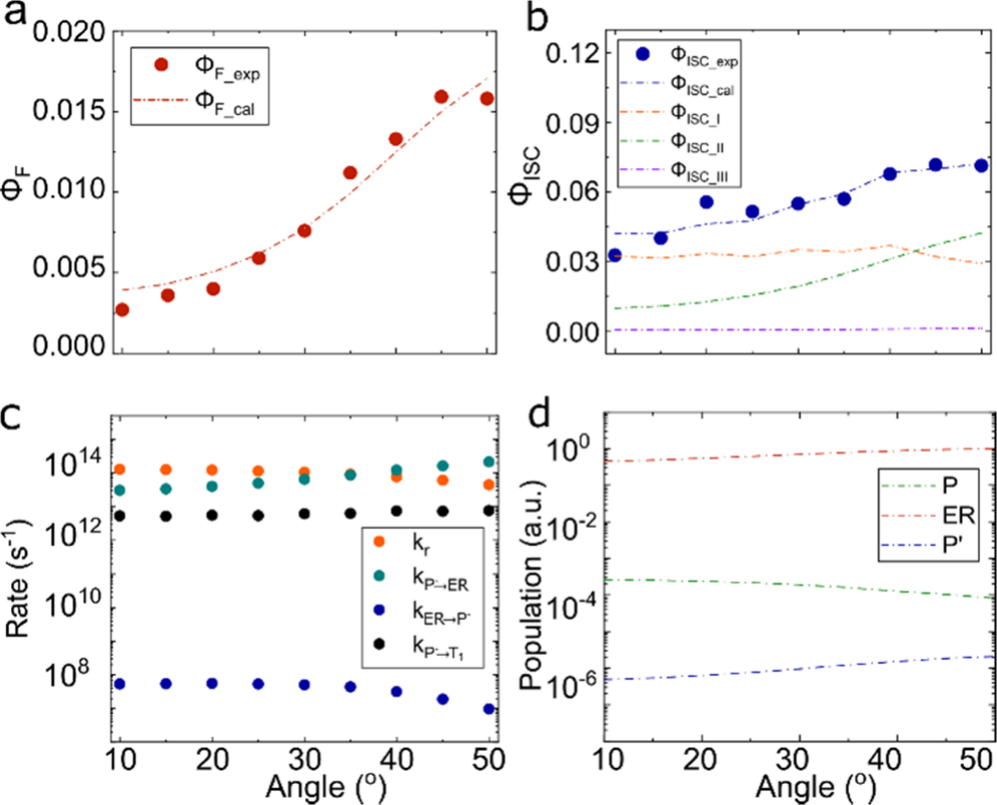
(a) Relaxation efficiency as a function of angle for cavity 4 in the prompt regime. The red dots are the experimental relaxation efficiencies (Φ_F_exp_). The red dashed line is the calculated prompt relaxation efficiency (Φ_F_cal_). (b) Relaxation efficiency as a function of angle for cavity 4 in the delayed regime. The blue dots are the experimental relaxation efficiencies (Φ_ISC_exp_). The blue dashed line is the calculated delayed relaxation efficiency (Φ_ISC_cal_). The orange, green, and purple dashed lines represent the contributions from *k*_P → T1_ (Φ_ISC_I_), *k*_ER → T1_ (Φ_ISC_II_) and *k*_P′ → T1_ (Φ_ISC_III_), respectively. (c) Fitted rates extracted from the experimental data for cavity 4. *k*_r_, *k*_P^–^ → ER_, *k*_ER → P^*–*^_, and *k*_P^–^ → T1_ are represented by orange, green, blue, and black dots, respectively. (d) Relative populations of P (shown as green dashed line), ER (shown as red dashed line), and P′ (shown as blue dashed line) as a function of angle (in logarithmic scale).

Figure 6a displays the Φ_F_ of cavity 4. The red dots show the experimental data, and the red dashed line represents the calculated Φ_F_ (the fitting parameters are given in Table S3). Here, the prompt efficiency describes the polariton emission when relaxation occurs via the exciton reservoir. In other words, the population first goes from P to ER, where the in-plane momentum randomizes, and then comes back to P^′^ from where emission occurs. The experimental results show that the relaxation efficiency increases with the excitation angle. They are well reproduced by the simulations, with the small difference that the experiments show a leveling off of the increase at 50° that is not as pronounced in the simulations.

The simulated efficiency of ISC (Figure 6b) is shown in a blue dashed line along with the experimental data (blue dots). Here, again, the experimental data are well reproduced by the simulation. The yield of ISC can be divided into three contributions. The first is a direct contribution from the initially excited P (orange dashed line, the first term in eq 6). The second and third contributions come into the picture after the initially excited P^–^ population has transferred to the exciton reservoir. From here, the population can relax to T_1_ (green dashed line, the second term in eq 6) or transfer to P′, from where it can relax to T_1_ (purple dashed line, the third term in eq 6). To understand the importance of the interaction between the delocalized polariton state and the localized triplet state, which is mainly guided by *k*_P^–^ → T1_, we fitted the relaxation efficiencies without involving the direct route (setting *k*_P^–^ → T1_ to zero). The results infer that the relaxation efficiencies cannot be fitted globally without the involvement of *k*_P^–^ → T1_ using this model (Figure S11), and we establish that there must be a direct transfer from P^–^ to the T_1_ state. Furthermore, Figure 6b suggests that the relative contribution to the total quantum yield of ISC depends on the angle of excitation. At low angles, the direct pathway is predominant. However, at higher excitation angles, the indirect route through the exciton reservoir increases significantly. It should be noted here that we cannot resolve if the direct pathway from P^–^ to the T_1_ state involves any intermediate steps (other than the ER). The presence or absence of intermediate steps can be explored by correlating the polariton decay with the build-up of the T_1_ population. However, the short time scales of the P^–^ decay make this a very difficult experiment to conduct, and this is therefore not further explored.

Before understanding the role of the exciton reservoir on the efficiencies, we will take a closer look on how the individual rates and populations govern the efficiencies. The angular dependency of the rate constants is depicted in Figure 6c. *k*_r_ and *k*_ER → P_^_–_^ decrease with angle due to a decrease in the photonic fraction of P^–^. In the case of *k*_ER → P^–^_, the reduced overlap between the emissive state of the weakly coupled molecule and the excited state of P^–^ play an additional role. On the other hand, *k*_P^–^ → ER_ increases significantly with angle due to the strong decrease in energy mismatch between the P^–^ branch and the exciton reservoir. In addition, the rate constant of the direct pathway (*k*_P^–^ → T1_) is not very sensitive on the excitation angle. This is because the overlap integral in eq S7 and Hop_mol_ counteract each other as a function of angle. Importantly, the rate of ISC from the P^–^ state (∼10^13^ s^–1^) is 5 orders of magnitude higher compared to that from the exciton reservoir (∼10^8^ s^–1^). This suggests that the delocalized polariton state efficiently decays to the localized triplet states if the involved energy levels are isoenergetic (eq S7). Energy relaxation from polaritonic states to lower-energy uncoupled charge transfer states has been previously observed.^57^ However, it has not previously been seen in conjunction with a spin flip. Furthermore, theoretical studies have shown that very fast relaxations from polaritonic toward isoenergetic or lower-energy states are possible.^58−60^ The polaritonic states are a discretization of a continuum. It is therefore interesting to assess the effect of the number of polaritonic states on the fitted rate constants. The only rate constant that shows such a dependence is *k*_ER → P^–^_, and it does so inversely linearly with the number of states used in the fitting procedure (Figure S12). When comparing rate constants from the exciton reservoir to the lower polaritonic branch, it is therefore advisable to normalize them with the angular step size taken or the sum over all angles.

The acquired populations of the states involved in the kinetic model are depicted in Figure 6d. The normalized populations of the P, ER, and P′ states are shown by green, red, and blue dashed lines, respectively (in logarithmic scale). The population of the P′ state at each angle of excitation is given in Figure S13. In the simulations, the population density of ER is by far the largest, and the epithet excitation reservoir is therefore an appropriate one. The relative population along with the rate constants can explain the delayed relaxation efficiencies. As already shown in Figure 6c, the transfer rate *k*_P^–^ → ER_ from the pumped polariton to the exciton reservoir is highly dependent on the excitation angle, and as a result, it dominates the population of the different states at different excitation angles. Thus, at higher angles, the relative population density of P reduces and the population density of ER increases, resulting in higher efficiencies from relaxation routes through the exciton reservoir (green dashed line, Figure 6b).

## Conclusions

In summary, this study conveys new quantitative insights into the rate of intersystem crossing in the strong exciton–photon coupling regime. Erythrosine B was encapsulated in an optical cavity, and the system was shown to enter the strong coupling regime. The angular dependence of the excitation and reflectivity spectra were used to obtain the quantum efficiencies of polariton emission and intersystem crossing. A model based on rate equations could fit the experimental data only if a direct route between the polaritonic state and the triplet state is present. The obtained rate constants suggests that the rate of intersystem crossing from polaritonic states can be 5 orders of magnitude higher than the corresponding rate observed for the bare molecule. This gives clear evidence that polaritonic states are prone to collapse to molecular localized isoenergetic states and the fact that the very short-lived polaritonic states are able to decay to noncoupled lower-energy states has been theoretically predicted for the case of singlet fission and photoreactions^58−60^ and indirectly observed when used to funnel energy in organic electronics.^57^ In light of the opportunities that transitions from polaritonic to molecular localized states offer within molecular photophysics/chemistry and organic electronics, we hope that the quantitative understanding of such interactions gained from this study will aid in the development of polariton-empowered devices.

## Supporting Information Available

Experimental methods, experimental results and analysis, and supplemental references (PDF)
