# Supplementary material for: Quantitative Investigation of the Rate of Intersystem Crossing in the Strong Exciton–Photon Coupling Regime
Source: J Am Chem Soc. 2023 Feb 22;145(9):5155–62. doi: 10.1021/jacs.2c11531 (PMC9999416; doi:10.1021/jacs.2c11531)
Supplement: Supplementary file 1 — ja2c11531_si_001.pdf [file ja2c11531_si_001.pdf]

Supporting Information for

A Quantitative Investigation of the Rate of Intersystem Crossing in the Strong Exciton-Photon Coupling Regime

Arpita Mukherjee[a], Johannes Feist[b] and Karl Börjesson[a]*

[a]Department of Chemistry and Molecular Biology, University of Gothenburg, Kemivägen 10, 412 96 Gothenburg (Sweden)

[b]Departamento de Física Teórica de la Materia Condensada and Condensed Matter Physics Center (IFIMAC), Universidad Autónoma de Madrid, Madrid, E-28049 (Spain)

*Corresponding Author: karl.borjesson@gu.se

Table of Contents

1. Experimental methods. 3

1.1 Film preparation. 3

1.2 Cavity preparation. 3

Table S1. Parameters for cavity preparation. 4

1.3 Steady state absorption, reflection, and emission spectroscopy. 4

1.4 Experimental set-up for angle resolved excitation measurements. 5

1.5 Modelling the angle-resolved reflectivity spectra. 6

2. Supporting experimental results and analysis. 7

2.1 Properties of the cavities 7

Table S2. Extracted parameters using a coupled harmonic oscillator model. 8

2.2 Emission from a cavity. 8

2.5 Angle-resolved excitation of the bare film. 9

2.3 Normalized PL Intensity as a function of angle. 9

2.4 Normalized excitation peak intensity of the cavities. 10

2.5 Absorbance of the cavities. 11

Table S3. Fitting parameters 11

2.6 Calculating the rate constants 12

2.7 Absorption of Ag mirror and bare cavity. 14

2.8 Connection between relaxation efficiency and emission quantum yield. 15

2.9 Relating intersystem crossing and delayed relaxation efficiency. 17

2.10 The influence of $k_{P^{'}\rightarrow T1}$. 17

2.11 The influence of the number of polaritonic states on the rate constants. 18

2.12 Population of the P´ states at all angles. 19

3. References 20

1. Experimental methods.

1.1 Film preparation.

Glass substrates (2.5 cm×2.5 cm) were immersed in alkaline solution (0.5 % of Hellmanex in distilled water) and sonicated there for 15 min. Afterwards the substrates were sonicated in water and ethanol, for 1 h, respectively. The cleaned glass substrates were dried in an oven overnight before cavity preparation. The ErB films were deposited on the cleaned glass substrates by spin-coating (Laurell Technologies WS-650).

15 wt% ErB/PVA film: 4.6 mg of ErB molecules were dissolved in 1 ml of water containing PVA (88% hydrolyzed, Acros Organics, 22 mg mL^{-1})[1-3]. The solution was filtered through a PHENEX syringe filter (pore size, 0.45 μm) and 250 μL of the solution was deposited by spin coating on a clean glass plate at various rpm (Table S1) for cavity preparation.

1.2 Cavity preparation.

The Fabry–Pérot cavities were built on glass substrates with a size of 2.5 × 2.5 cm^2. The Ag mirrors were fabricated by vacuum sputtering deposition (HEX, Korvus Technologies). A 100 nm thick Ag film was sputtered on top of the cleaned glass substrate and the molecules were then deposited by spin-coating (Laurell Technologies WS-650) on top of the Ag layer. The spin-coating speed was varied from 1200 to 3000 rpm to achieve different film thicknesses. A semitransparent 30 nm thick Ag film was then sputtered on top of the molecular film to complete the cavities. The typical cavity configuration and detailed parameters for different cavities are listed in Table 1.

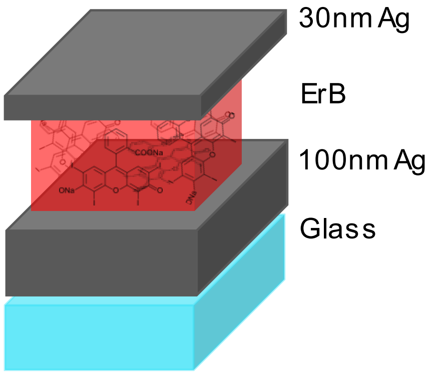

Scheme S1. A typical Fabry–Pérot cavity used to study strong light-matter interactions in this study (glass support/100 nm Ag/ErB-PVA film/30 nm Ag).

Table S1. Parameters for cavity preparation.

Sample	Solution for preparation		Spin-coating speed
	PVA	ErB	(rpm)
Cavity 1	26 mg/ mL	4.6 mg/ mL	3000
Cavity 2	26.3 mg/ mL	4.7 mg/ mL	2000
Cavity 3	26.3 mg/ mL	4.5 mg/ mL	1800
Cavity 4	26.3 mg/ mL	4.7 mg/ mL	1500
Cavity 5	26 mg/ mL	4.6 mg/ mL	1200

1.3 Steady state absorption, reflection, and emission spectroscopy.

Steady state absorption spectra of bare films and cavities were recorded on a Perkin Elmer LAMBDA 950 spectrometer. Steady-state angle-dependent reflectance spectra were measured in 2 degree intervals using a spectrophotometer equipped with a small-angle specular reflectance accessory (Lambda 950, Perkin Elmer). The spectra were measured relative to a standard reflectance mirror using a horizontal Glan-Taylor polarizer. The reflectance spectra were collected in TE mode using the polarizer. The absorbance of these cavities were determined by using $Abs = log(1/R)$, assuming that transmission and scattering by the cavities are zero. The quality factor ($Q = \omega_r/\Delta\omega$) of an empty cavity (glass support/100 nm Ag/PVA film/30 nm Ag) was around 20 (Figure S1).

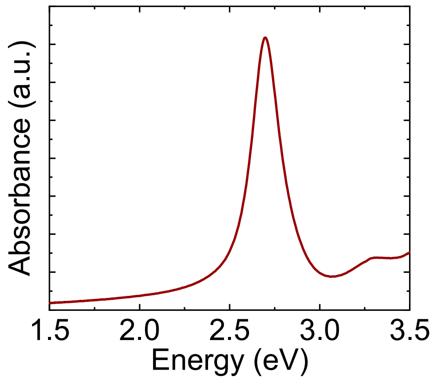

Figure S1. Absorbance of bare cavity (glass support/100 nm Ag/PVA film/30 nm Ag). The quality factor is 20.

Steady state emission spectra of the films and the cavities were measured on an Edinburgh Instruments FLS 1000 spectrofluorometer with a Xenon lamp as excitation source. Angular resolved emission spectra of the cavities were measured on the same instrument using a fiber induced angle-resolved platform.

1.4 Experimental set-up for angle resolved excitation measurements.

Steady-state excitation spectra were measured with a spectrofluorometer (FLS1000, Edinburgh Instruments). The optical properties of the cavities were studied in front-face geometry. Excitation and the emission paths were fiber-coupled to the rotating arm of a goniometer. The experimental setup is shown in Scheme S2. During the measurement, the excitation angle was changed in 5° interval, and the emission angle was fixed. The excitation was polarized in the TE plane. Due to the experimental geometry, the smallest angle at which the emission was collected is around 20°. The emission was monitored about 20 nm towards a longer wavelength as compared to the P^{-} emission. To probe prompt and delayed spectra, a µs-pulsed lamp was used (Edinburgh Instruments). The "prompt" spectra were recorded at times when the lamp was on, and the "delayed" spectra were recorded 210 µs after the lamp flash was over (gate time was 1000 µs).

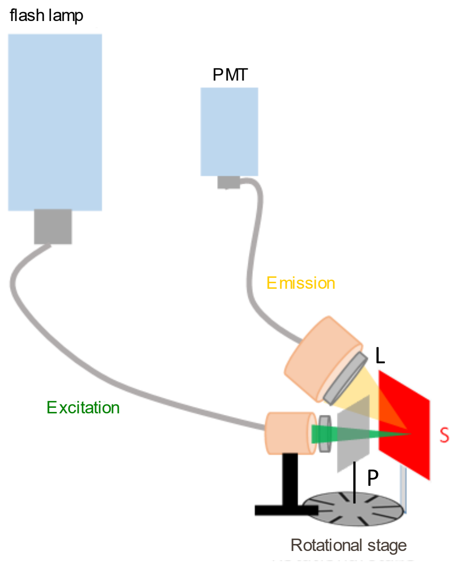

Scheme S2. Angle resolved excitation spectra setup (S is the sample, P is the polarizer). The flash lamp (pulse period is in microsecond) operates as the excitation source and the emission is collected by a PMT.

1.5 Modelling the angle-resolved reflectivity spectra.

In order to fit and extract parameters from angle-resolved reflectivity spectra, we used the coupled harmonic oscillators (CHO) model. The coupling is described by a 2 × 2 Hamiltonian matrix describing the interaction between a photon and an exciton inside the cavity, as shown below:

$$\begin{pmatrix} E_c(k_{||}) & V_A \\ V_A & E_x(k_{||}) \end{pmatrix} \begin{pmatrix} \alpha \\ \beta \end{pmatrix} = E \begin{pmatrix} \alpha \\ \beta \end{pmatrix} \quad \text{(S1)}$$

Where E_c is the cavity energy, E_x is the exciton energy, and V_A is the coupling strength (corresponding to a Rabi splitting of $\hbar\Omega_R = 2V_A$ at resonance). α and β are the Hopfield coefficients. Diagonalization of this Hamiltonian gives rise to the eigenvalues of the Hamiltonian that represent the polariton energies:

$$E_{P+/P-} = \frac{1}{2}(E_x + E_c) \pm \sqrt{V_A^2 + \frac{1}{4}(E_x - E_c)^2} \quad \text{(S2)}$$

here $E_c(\theta)$ is the energy dispersion of the cavity, described by

$$E_c(\theta) = E_c(0)(1 - ({}^{sin\theta}/_{n_{eff}})^2)^{-1/2} \quad \text{(S3)}$$

Where $E_c(0)$ is the cavity energy at angle zero, θ is the angle of incidence, and n_{eff} is the refractive index of the medium.

Here, $E_c(0) = E_x + \Delta$, where Δ is the detuning parameter.

2. Supporting experimental results and analysis.

2.1 Properties of the cavities

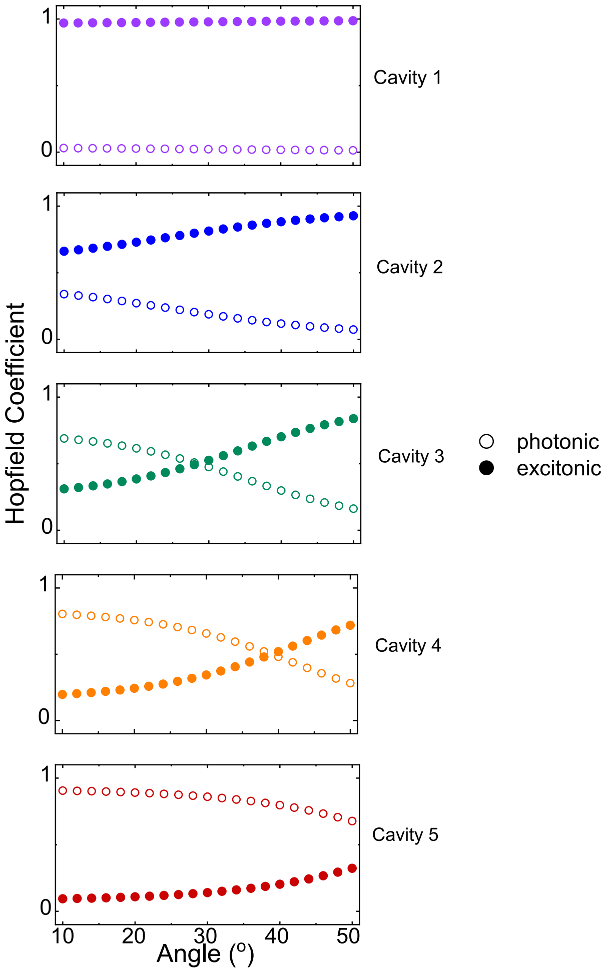

Figure S2. Hopfield coefficients of P$^-$ of the cavities.

Table S2. Extracted parameters using a coupled harmonic oscillator model. The FWHM of the molecular transition is 180 meV (Figures 1b), and the FWHM of an empty cavity is 140 meV. These values are smaller than $2V_a$, thus the cavities are in the strong coupling regime.

Cavity 1	Cavity 2	Cavity 3	Cavity 4	Cavity 5
$E_c(0)$ = 2.84 eV	$E_c(0)$ = 2.37 eV	$E_c(0)$ = 2.19 eV	$E_c(0)$ = 2.1 eV	$E_c(0)$ = 1.93 eV
E_x = 2.3 eV	E_x = 2.3 eV	E_x = 2.3 eV	E_x = 2.3 eV	E_x = 2.3 eV
n_{eff} = 1.75	n_{eff} = 1.62	n_{eff} = 1.53	n_{eff} = 1.54	n_{eff} = 1.59
V_a = 0.10 eV	V_a = 0.12 eV	V_a = 0.12 eV	V_a = 0.12 eV	V_a = 0.13 eV

2.2 Emission from a cavity.

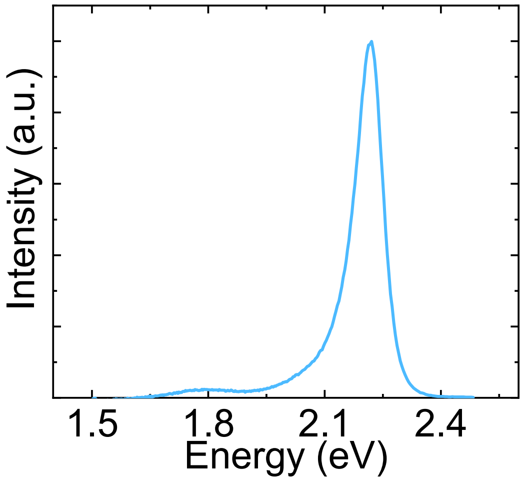

Figure S3. Emission from Cavity 1 at 0°.

2.5 Angle-resolved excitation of the bare film.

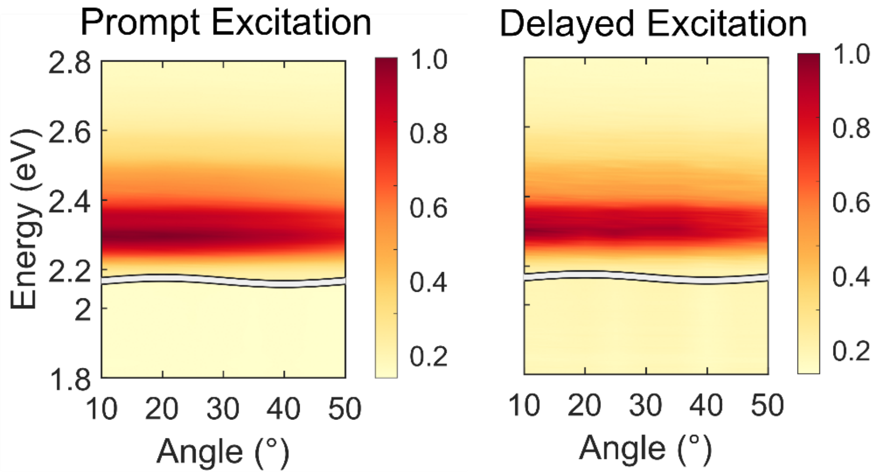

Figure S4. Excitation spectra of a film as a function of excitation angle in the prompt and delayed regimes (collected in TE mode). The excitation energy was varied from 2.8 eV to 2.16 eV first and then from 2.09 eV to 1.8 eV, to avoid the scattering from the laser at the emission wavelength (2.12 eV). The two graphs are very similar, suggesting that the differences in the prompt and delayed excitation spectra in Figure 3 are not due to the molecule used.

2.3 Normalized PL Intensity as a function of angle.

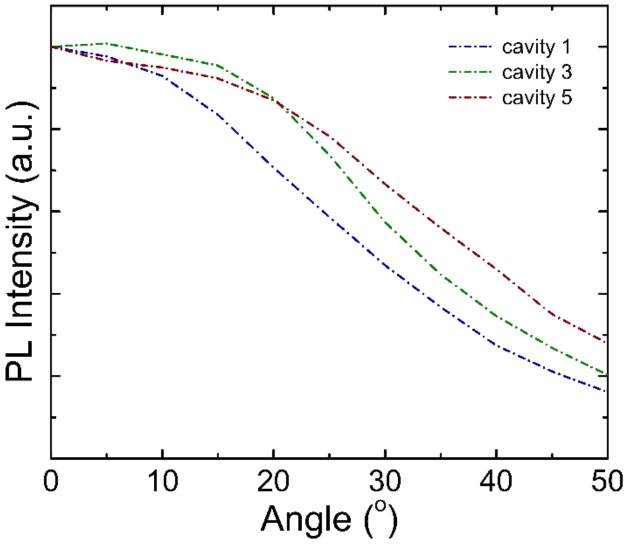

Figure S5. Integrated emission from Cavity 1 (blue dashed line), 3 (green dashed line) and 5 (red dashed line) as a function of angle.

2.4 Normalized excitation peak intensity of the cavities.

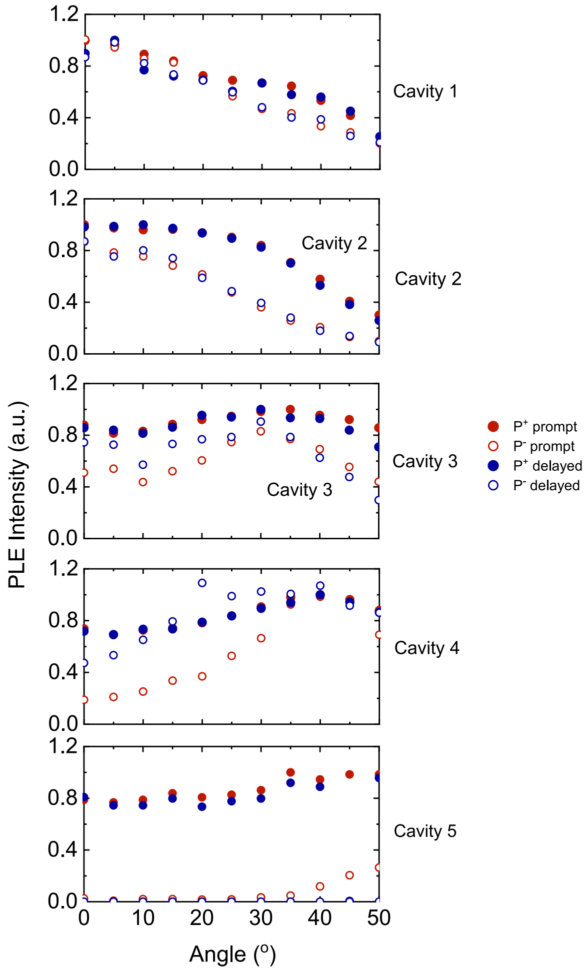

Figure S6. The extracted and normalized excitation peak counts (here the peaks are normalized at the maximum intensity). For the blue detuned cavities (1 and 2) the P^+ and P^- intensity remains the same in the prompt and delayed regimes. For the red detuned cavities (3,4 and 5), the P^+ intensity remains similar, but the P^- intensity differs in the prompt and delayed regimes.

2.5 Absorbance of the cavities.

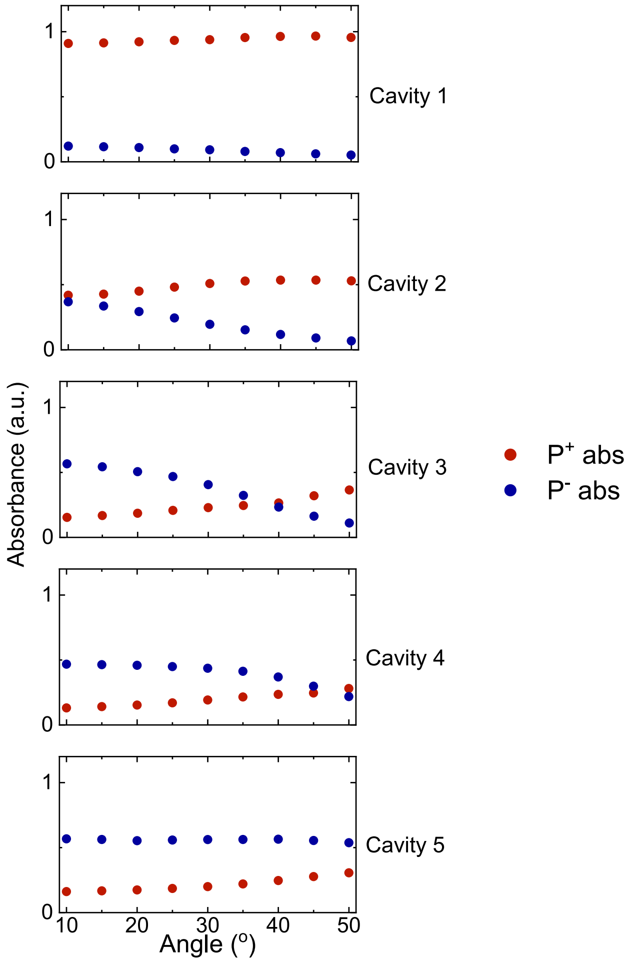

Figure S7. The absorbance peak counts of the cavities, interpolated in 5 degree interval from the absorbance taken in 2 degree interval.

Table S3. Fitting parameters from the calculations.

Parameters	Cavity 4
C_1	1.10×10^{14}
C_2	2.54×10^{4}
C_3	1.09×10^{12}

2.6 Calculating the rate constants

The radiative decay k_r of the polariton is proportional to the photonic fraction Hop_{ph} of P^-, and inversely proportional to the quality factor of the cavity. For a metal clad cavity, we consider it to be:

$$k_r(\theta) = \frac{Ec}{\hbar \cdot \mathrm{QF}} \cdot Hop_{ph}(\theta) \qquad \text{(S4)}$$

Where Ec is the energy of a bare cavity and QF equals to the cavity quality factor (=20). Using these values, the rate is $1.6 \cdot 10^{14}$ s^{-1} (without incorporating $Hop_{ph}(\theta)$). The polariton to exciton reservoir transfer rate, $k_{P^- \to ER}$, is estimated as proportional to the energetic penalty for endothermically repopulating the exciton reservoir.[4] Here we consider the P^- state as a delta function in energy scale by assuming that the polariton energy is well-defined. The ER bandwidth is taken into account by an integral that is scaled with an exponential factor, which includes the polariton–exciton energy separation. The exponential function is set to 1 for the energies where the transfer is exothermic. The rate constant is further scaled with the excitonic fraction Hop_{mol} of the P^- branch as $k_{P^- \to ER}$ signifies the transfer to the exciton reservoir:

$$k_{P^- \to ER}(\theta) = C_1 \cdot Hop_{mol}(\theta) \cdot \int_0^{\infty} Abs_{film}(E)\, e^{-(E - E_{P^-}(\theta))/(k_B T)} dE \qquad \text{(S5)}$$

where E is the energy scale over which the absorbance is measured, E_{P^-} is the energy maximum of P^- at each angle (θ), and $k_B T$ is the energy corresponding to room temperature.

We consider the transfer between the exciton reservoir and P´ states by a radiative pumping mechanism.[5] For small organic molecules this is often the case due to their relatively large Stokes shift.[6] The rate is therefore proportional to the overlap between the emission intensity of the bare film, $Em_{film}(E)$ and the P^- absorption, $Abs_{P^-}(E, \theta)$. It also depends on Hop_{ph} as the transfer is photonically mediated.

$$k_{ER \to P^-}(\theta) = C_2 \cdot Hop_{ph}(\theta) \cdot \int_0^{\infty} Abs_{P^-}(E, \theta)\, Em_{film}(E)\, dE \qquad \text{(S6)}$$

Transfer from ER to the triplet state, $k_{ER \to T1}$, was set to the literature value of ISC in a concentrated film of ErB ($k_{ER \to T1} = 5.7 \cdot 10^8$ s^{-1}),[7] and k_{nr} corresponds to the non-radiative decay of the exciton reservoir. This value can be approximated with the non-radiative decay of ErB outside the cavity. At the used concentrations, the emission quantum yield of ErB is low, and the non-radiative decay can therefore be approximated by the inverse excited state lifetime. To probe this value, the emission decay from a neat film was taken, but the lifetime

was below the time resolution of our TCSPC instrument. k_{nr} was therefore set to the time resolution of the instrument, which is about 100 ps. The set value of k_{nr} can therefore be regarded as a lower boundary. We assume that k_{nr} is constant with excitation wavelength. Previous work has shown that the polariton emission lifetime, and thus presumably the non-radiative rate, can be dependent on excitation wavelength.[8] We therefore explored how sensitive our conclusions are on the set value of k_{nr}. The direct pathway from P^- to T_1 becomes enhanced as k_{nr} increases and vice versa. Relatively good fits can be performed with k_{nr} values in the $1 \cdot 10^{11}$ - $8 \cdot 10^{9}$ s^{-1} range. However, most importantly, for all cases where physically sound fits can be achieved (i.e., positive fitting coefficients), does the direct pathway from P^- to T_1 significantly contribute to the yield of ISC.

We assume that transfer from P^- to the triplet state (T_1) depends on the energy overlap between these two states. Then, $k_{P^- \rightarrow T1}$ becomes proportional to the energy overlap between the absorption of the triplet state (Abs_T) and P^-. The triplet state energy cannot readily be experimentally obtained but an estimate of it was made by first assuming that the spectral envelope of the triplet and singlet states are approximately the same. It was further assumed that the reorganization energy on the singlet and triplet surfaces are about the same. In practice was the T_1 energy constructed by translating the S_1 absorbance to an energy, one Stokes shift higher as compared to the phosphorescence maximum (Figure S8).

$$k_{P^- \rightarrow T_1}(\theta) = C_3 \cdot Hop_{mol} \cdot \int_0^{\infty} Abs_T(E)\, Abs_{P-}(E,\theta) dE \qquad \text{(S7)}$$

It should be noted here, that we cannot exclude the possibility that the transfer from P^- to T_1, is mediated by an optically dark short-lived state, such as an excimeric state, as the observed kinetics would be the same.

When fitting the experimental data to the rate equation model (equations 5 and 6), C_1, C_2, C_3 was used as global fitting parameters.

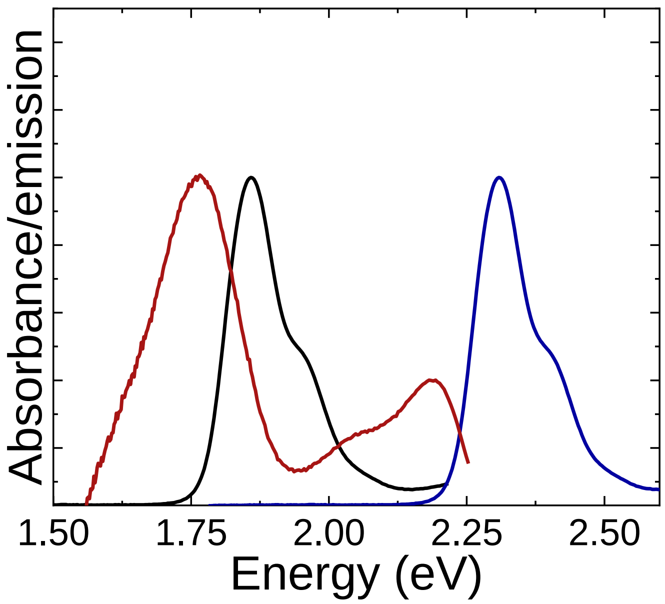

Figure S8. Absorbance (blue line) spectrum of a 15 wt% ErB film in a PVA polymer matrix (glass support/ErB-PVA film). Also shown is the approximated energy of the T_1 state (black line), and the emission from a half cavity (red line; glass support/100 nm Ag/ErB-PVA film).

2.7 Absorption of Ag mirror and bare cavity.

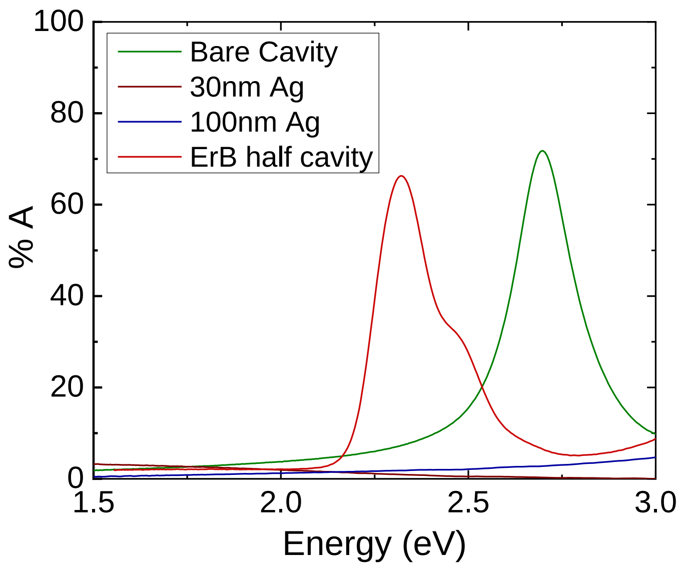

Figure S9. Absorption of bare cavity (green), half cavity (red), 100 nm Ag mirror (blue) and 30 nm (burgundy) Ag mirror. The absorption was calculated as 1-T-R.

2.8 Connection between relaxation efficiency and emission quantum yield.

The relaxation efficiency is proportional to the population of a state before emission. As a result, it is also proportional to the emission quantum yield (QY) of the system and to connect the relaxation efficiency to the QY we measured the emission QY of Cavity 4 to 0.8% using an integrating sphere (Edinburgh Instruments). To mention, the relaxation efficiencies were measured in the prompt and delayed regime. The prompt and delayed parts of the total QY of emission was extracted as follows:

1. The excitation spectra were collected at 20° emission angle using a μs-pulsed lamp, which was incorporated into a commercial spectrofluorometer (FLS1000 Edinburgh Instruments). The excitation wavelength was selected using the double monochromator of the spectrofluorometer, and all recorded spectra are corrected. This lamp has a temporal resolution of a couple of microseconds, but it afterburns for an additional few 10s of microseconds. The prompt emission was taken as the emission during the pulse duration. It was collected using a gate time of 30 μs. The delayed emission was collected 210 μs after time-zero of the flash (this long delay was applied in order to ensure no residual afterburning of the lamp) and the corresponding gate time was 1000 μs. The long delay time was to avoid effects from afterburning of the lamp flash.

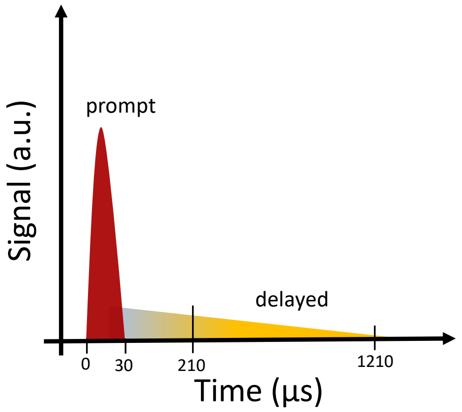

Scheme S3. Schematic of prompt and delayed signal as a function of time. The red region represents the prompt signal the yellowish orange shows the delayed region.

2. Next, we calculate the prompt and delayed parts of the QY. The total QY is considered to be proportional to the sum of the prompt and delayed contributions. The prompt emission counts (x) is proportional to the counts collected from 0 μs to 30 μs (0° excitation and 20° emission; deep red region in Scheme S3). The get the delayed emission counts, the 210 μs to 1210 μs

area was first integrated (yellowish orange region in Scheme S3). This data was then extrapolated to 30 μs. To do that, we consider first order decay kinetics and the delayed counts from 210 μs to 1210 μs = y

As a result,

$$A\int_{210}^{1210} e^{-kt}dt = y \tag{S8}$$

Here, k = 1/432 μs, which was calculated from the phosphorescence decay of an ErB film.

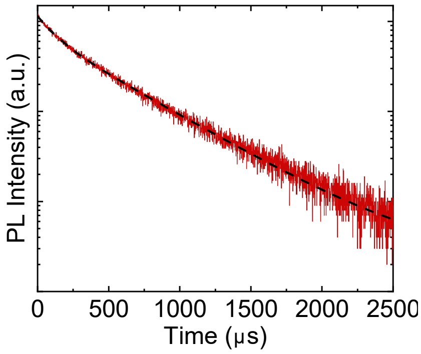

Figure S10. Phosphorescence decay of an ErB film.

After determining A, we calculated the total delayed counts from 30 μs to 1210 μs, which is considered as z.

$$z = A\int_{30}^{1210} e^{-kt}dt \tag{S9}$$

With this at hand we can calculate the prompt and delayed contribution to the total QY.

$$QY_{prompt} = \frac{x}{x+z}QY_{total} \tag{S10}$$

$$QY_{delayed} = \frac{z}{x+z}QY_{total} \tag{S11}$$

After the relaxation efficiencies at 0° excitation and 20° emission had been determined, the efficiencies from other excitation angles were calculated from the values in Figure 4.

2.9 Relating intersystem crossing and delayed relaxation efficiency.

Equation 6 expresses the delayed relaxation efficiency (φ_{ISC}) as a function of rate constants. In section SI 2.5 on the other hand, we measure the delayed emission efficiency. To emit a photon from the triplet state, reverse intersystem crossing followed by polariton emission needs to occur. The triplet state, exciton reservoir, and the polaritonic states are in dynamic equilibrium with each other. However, in our simplified model (Figure 5) we assume that non-radiative relaxation from the triplet state is much faster than reverse intersystem crossing. Thus, from a population perspective, the small yield of repopulation of P^- from the triplet state does not affect the population density at constant illumination (although we use the photons emitted from this event in our experiments). Thus, we can take the literature ratio between the ISC and the RISC processes ($\frac{\varphi_{ISC}}{\varphi_{RISC}}$) $= 5$ in order to scale the measured $QY_{delayed}$ with φ_{ISC} that is used in Equation 6.[9] Furthermore, as emission is constantly monitored in the same angle, this value should be constant throughout our dataset, no matter that measurements were conducted in the strong exciton-photon coupling regime.

2.10 The influence of $k_{P^- \to T1}$.

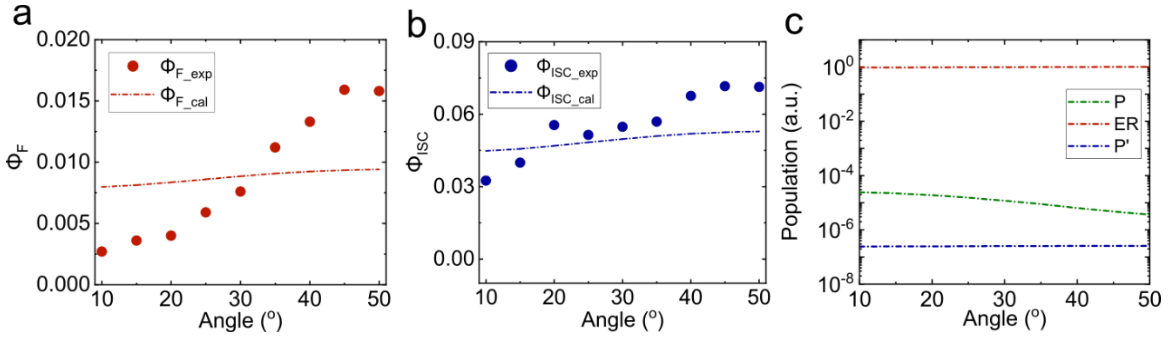

Figure S11. a) The relaxation efficiency as a function of angle for cavity 4 in the prompt regime when setting $k_{P^- \to T1} = 0$ during the fit. The dark red dots are the experimental relaxation efficiencies. The dark red dashed line are the calculated prompt relaxation efficiencies. **b)** The relaxation efficiency as a function of angle for cavity 4 in the delayed regime when setting $k_{P^- \to T1} = 0$ during the fit. The blue dots are the experimental relaxation efficiencies. The blue dashed line are the calculated delayed relaxation efficiencies. The delayed and prompt components were fitted globally. **c)** Relative populations at steady state conditions.

2.11 The influence of the number of polaritonic states on the rate constants.

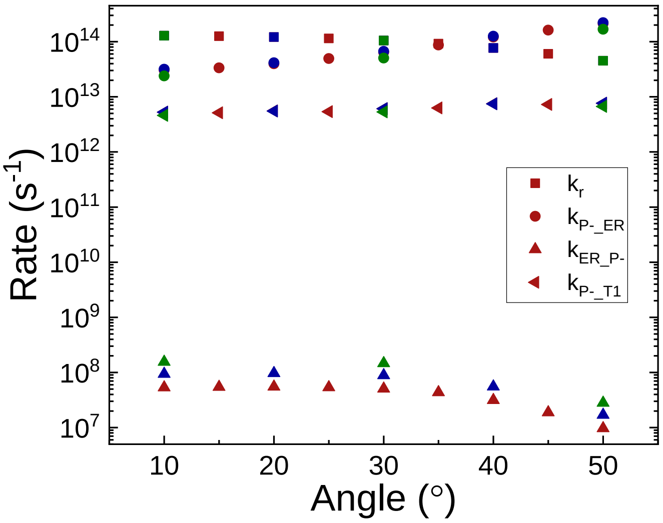

Figure S12. The fitted rate constant extracted from the experimental data for Cavity 4. The color coding shows the number of angles (thus polaritonic states) used in the fit, where red denotes all 9 angles (thus showing the same data as in Figure 6c), blue denotes 5 angles (every 10 degrees), and green denotes 3 angles (10, 30, and 50 degrees). Note that some points are overlayed and can therefore be difficult to see. The only rate constant that depends on the number of polaritonic states is $k_{ER\rightarrow P^-}$. However, the rate of depopulation from the exciton reservoir is constant. In other words, the sum of $k_{ER\rightarrow P^-}$ over all angles is constant, $\sum k_{ER\rightarrow P^-}$ $=3.7\cdot 10^8$ s^{-1}, $\sum k_{ER\rightarrow P^-}=3.5\cdot 10^8$ s^{-1}, and $\sum k_{ER\rightarrow P^-}=3.3\cdot 10^8$ s^{-1} when using 9, 5, and 3 polaritonic states in the fit, respectively. Another way of comparing this rate constant for a different discretization of the polaritonic states would be to involve the step size. This would be a physically sound method, but could give errors in the limit of a very small number of angles (comparing 5 and 3 polaritonic states, the step size relates as 20:10, but the number of states as 3:5).

2.12 Population of the P´ states at all angles.

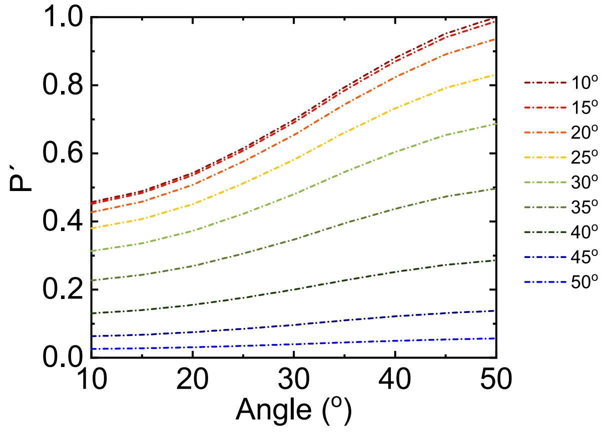

Figure S13. Relative population of P´ states from 10° to 50° angles.

3. References

1. Pant, D. D.; Bhagchandani, C. L.; Pant, K. C.; Verma, S. P., Aggregation in xanthene dyes, exciton emission and phosphorescence enhancement. *Chem. Phys. Lett.* **1971,** *9* (6), 546-547.
2. Joshi, N. B.; Pant, D. D., Effect of aggregation on the radiative (T1 → S0) and nonradiative (T1⇝S0 and S1⇝T1) transitions in xanthene dyes. *J. Lumin.* **1976,** *14* (1), 1-8.
3. Stomphorst, R. G.; van der Zwan, G.; van Zandvoort, M. A. M. J.; Sieval, A. B.; Zuilhof, H.; Vergeldt, F. J.; Schaafsma, T. J., Spectroscopic Study of Erythrosin B in PVA Films. *J. Phys. Chem. A* **2001,** *105* (17), 4235-4240.
4. Groenhof, G.; Climent, C.; Feist, J.; Morozov, D.; Toppari, J. J., Tracking Polariton Relaxation with Multiscale Molecular Dynamics Simulations. *J. Phys. Chem. Lett.* **2019,** *10* (18), 5476-5483.
5. Coles, D. M.; Somaschi, N.; Michetti, P.; Clark, C.; Lagoudakis, P. G.; Savvidis, P. G.; Lidzey, D. G., Polariton-mediated energy transfer between organic dyes in a strongly coupled optical microcavity. *Nat. Mater.* **2014,** *13* (7), 712-719.
6. Hulkko, E.; Pikker, S.; Tiainen, V.; Tichauer, R. H.; Groenhof, G.; Toppari, J. J., Effect of molecular Stokes shift on polariton dynamics. *J. Chem. Phys.* **2021,** *154* (15), 154303.
7. Stranius, K.; Hertzog, M.; Börjesson, K., Selective manipulation of electronically excited states through strong light–matter interactions. *Nat. Commun.* **2018,** *9* (1), 2273.
8. Georgiou, K.; Jayaprakash, R.; Askitopoulos, A.; Coles, D. M.; Lagoudakis, P. G.; Lidzey, D. G., Generation of Anti-Stokes Fluorescence in a Strongly Coupled Organic Semiconductor Microcavity. *ACS Photonics* **2018,** *5* (11), 4343-4351.
9. Lettinga, M. P.; Zuilhof, H.; van Zandvoort, M. A. M. J., Phosphorescence and fluorescence characterization of fluorescein derivatives immobilized in various polymer matrices. *Phys. Chem. Chem. Phys* **2000,** *2* (16), 3697-3707.
